# The Multidisciplinary Heart Team in Cardiovascular Medicine: Current Role and Future Challenges

**DOI:** 10.1016/j.jacadv.2022.100160

**Published:** 2023-01-11

**Authors:** Wayne B. Batchelor, Saif Anwaruddin, Dee Dee Wang, Elizabeth M. Perpetua, Ashok Krishnaswami, Poonam Velagapudi, Janet F. Wyman, David Fullerton, Patricia Keegan, Alistair Phillips, Laura Ross, Brij Maini, Gwen Bernacki, Gurusher S. Panjrath, James Lee, Jeffrey B. Geske, Fred Welt, Prashanth D. Thakker, Anita Deswal, Ki Park, Michael J. Mack, Martin Leon, Sandra Lewis, David Holmes

**Affiliations:** a ACC Interventional Cardiology Section Leadership Council, Inova Heart and Vascular Institute, Falls Church, Virginia, USA; b Division of Cardiology, ACC Interventional Section Leadership Council, St. Vincent Hospital, Worcester, Massachusetts, USA; c ACC Cardiovascular Imaging Section Leadership Council, Center for Structural Heart Disease, Henry Ford Hospital, Detroit, Michigan, USA; d ACC Cardiovascular Team Section Leadership Council, Empath Health Services, Seattle, Washington, USA; e Seattle Pacific University, School of Health Sciences and School of Nursing, Seattle, Washington, USA; f Division of Cardiology, ACC Geriatric Cardiology Section Leadership Council, Kaiser Permanente San Jose Medical Center, San Jose, California, USA; g Division of Geriatrics, Stanford University School of Medicine, Stanford, California, USA; h Division of Cardiology, ACC Early Career Professionals Section Leadership Council, University of Nebraska Medical Center, Omaha, Nebraska, USA; i ACC Cardiovascular Team Section Leadership Council, Center for Structural Heart Disease, Henry Ford Health System, Detroit, Michigan, USA; j Division of Cardiothoracic Surgery, Department of Surgery, ACC Cardiac Surgery Team Section Leadership Council, University of Colorado School of Medicine, Aurora, Colorado, USA; k Division of Cardiology, ACC Cardiovascular Team Section Leadership Council, Emory Structural Heart and Valve Center, Emory University Hospital Midtown, Atlanta, Georgia, USA; l ACC Cardiac Surgery Team Section Leadership Council, The Heart, Vascular, and Thoracic Institute, Cleveland Clinic, Cleveland, Ohio, USA; m ACC Interventional Cardiology Section Leadership Council, Park Nicollet Heart and Vascular Center, St. Louis Park, Massachusetts, USA; n Charles E. Schmidt College of Medicine, Florida Atlantic University, Delray Medical Center, Delray Beach, Florida, USA; o Cardiovascular Division, Department of Medicine, ACC Geriatric Section Leadership Council, Cambia Palliative Care Center of Excellence, University of Washington, Seattle, Washington, USA; p Veterans Administration of Puget Sound, University of Washington School of Medicine, Seattle, Washington, USA; q ACC Heart Failure and Transplant Section Leadership Council, George Washington University School of Medicine and Health Sciences, Washington, DC, USA; r Department of Cardiovascular Medicine, ACC Cardiovascular Imaging Section Leadership Council, Mayo Clinic, Rochester, Minnesota, USA; s Division of Cardiovascular Medicine, ACC Interventional Cardiology Leadership Council, University of Utah Health Sciences Center, Salt Lake City, Utah, USA; t Cardiovascular Division, Department of Medicine, ACC Fellows in Training Section Leadership Council, Washington University, Saint Louis, Missouri, USA; u Division of Internal Medicine, Department of Cardiology, ACC Cardio-Oncology Leadership Council, The University of Texas MD Anderson Cancer Center, Houston, Texas, USA; v Division of Cardiovascular Medicine, ACC Interventional Cardiology Section Leadership Council, University of Florida College of Medicine, Malcom Randall VA Medical Center, Gainesville, Florida, USA; w Division of Cardiothoracic Surgery, Department of Surgery, ACC Cardiac Surgery Team Section Leadership Council, Baylor Scott and White Health, Dallas, Texas, USA; x Division of Cardiology, Department of Medicine, ACC Leon Center Leadership Council, Columbia University Irving Medical Center, New York, New York, USA; y ACC Section Steering Committee, Legacy Medical Group Cardiology, Portland, Oregon, USA; z Department of Cardiovascular Medicine, Mayo Clinic, Rochester, Minnesota, USA

**Keywords:** multidisciplinary heart team, structural heart disease, team-based care

## Abstract

Cardiovascular multidisciplinary heart teams (MDHTs) have evolved significantly over the past decade. These teams play a central role in the treatment of a wide array of cardiovascular diseases affecting interventional cardiology, cardiac surgery, interventional imaging, advanced heart failure, adult congenital heart disease, cardio-oncology, and cardio-obstetrics. To meet the specific needs of both patients and heart programs, the composition and function of cardiovascular MDHTs have had to adapt and evolve. Although lessons have been learned from multidisciplinary cancer care, best practices for the operation of cardiovascular MDHTs have yet to be defined, and the evidence base supporting their effectiveness is limited. This expert panel review discusses the history and evolution of cardiovascular MDHTs, their composition and role in treating patients across a broad spectrum of disciplines, basic tenets for successful operation, and the future challenges facing them.

## Highlights

• The MDHT team plays a central role in the treatment of a broad array of complex diseases across multiple cardiovascular medicine subspecialties.
• There is no widely accepted standard on the composition and function of cardiovascular MDHTs and the processes for rendering treatment decisions.
• Key principles exist for the effective operationalization of the MDHT.
• MDHTs must adapt to the specific needs of the patient and changing landscape of cardiovascular therapies.
• Further research is required to determine best practices for cardiovascular MDHTs and their impact on team efficiency and satisfaction and patient outcomes.

## Brief history of the multidisciplinary heart team

Driven by a need to bring together multiple specialists to render highly individualized treatment protocols, cancer care and solid organ transplantation programs have used multidisciplinary teams for decades.^1^^,^^2^ In fact, multidisciplinary team care is now widely accepted as the preferred model for many cancer treatments.^1^ The use of a multidisciplinary heart team (MDHT) in cardiovascular medicine has origins in clinical trials comparing myocardial revascularization strategies for coronary artery disease (CAD)^3^, ^4^, ^5^, ^6^ and subsequent studies evaluating transcatheter aortic valve replacement (TAVR) for aortic stenosis (AS)^7^^,^^8^ which required discussion among a cardiac surgeon, interventional cardiologist, and interventional imaging physician. In these circumstances, the primary objective of the MDHT was to form consensus on treatment decisions in complex patients for whom both surgical and percutaneous interventions were available, each with its own corresponding risks and benefits.

The use of a cardiovascular MDHT was first applied in the SYNTAX (Synergy Between PCI with Taxus and Cardiac Surgery) trial, in which a cardiac surgeon and interventional cardiologist were required to document clinical equipoise between coronary artery bypass graft surgery (CABG) and percutaneous coronary intervention (PCI).^5^ Official clinical endorsement of the MDHT was later issued by the 2014 European Society of Cardiology/European Association for Cardio-thoracic Surgery revascularization guidelines, which put forth a Class IC recommendation for MDHT assessment in patients for whom decision-making was complex and/or not covered by an institutional protocol.^9^ Since then, the MDHT has received Class I recommendations in both the United States and European guidelines for valvular heart disease (VHD) and for patients with left main and multivessel CAD who are being considered for PCI or CABG.^10^, ^11^, ^12^ These recommendations have been further codified by the Centers for Medicare & Medicaid Services (CMS) National Coverage Decisions for TAVR and transcatheter mitral valve edge-to-edge repair, for which MDHT evaluation has become a requirement for payment.^13^^,^^14^ Beyond myocardial revascularization decisions (PCI vs CABG) and VHD, there are multiple other fields of cardiovascular medicine that depend on MDHT input, including advanced heart failure (HF) and cardiac transplantation, adult congenital heart disease (ACHD), cardio-oncology, cardio-obstetrics, and geriatric cardiology. Therefore, contemporary MDHTs must continually adapt to the changing needs and increasing number of patients considered for advanced therapies that span broad cardiovascular disciplines.

## Composition and structure of the MDHT

Although there is no universally accepted structure for the cardiovascular MDHT, our conceptual framework of the MDHT places the patient at the top, aided by a team of health care professionals who work collaboratively throughout the patient’s continuum of care (Central Illustration). Core team members are typically comprised of the key health care personnel involved in the routine evaluation and treatment of the patient during various phases of care. Involvement of the patient’s family and/or close friend(s) may add perspective, empower the patient, enhance informed consent, and improve overall patient satisfaction.^15^ Given that treatment decisions require a firm understanding of procedural risks and benefits, quality of life/functional status, patient expectations, and health values, this additional input is often essential, especially in older adults with frailty and/or cognitive impairment. Depending on the clinical scenario and phase of care, other extended MDHT members (ie, pulmonary medicine specialists, vascular medicine/surgical specialists, neurologists, nephrologists, and others) may provide valuable input. Although this construct of MDHT structure and composition (Central Illustration) provides a useful conceptual framework, there is no widely accepted standard, as MDHTs may vary both within and across cardiovascular subspecialties, and the distinction between core and extended team members is somewhat arbitrary. However structured, successful MDHTs must possess the collective expertise necessary to manage a wide range of complex disease scenarios and flexible enough to adjust to the unique needs of the individual patient.

**Central Illustration:**
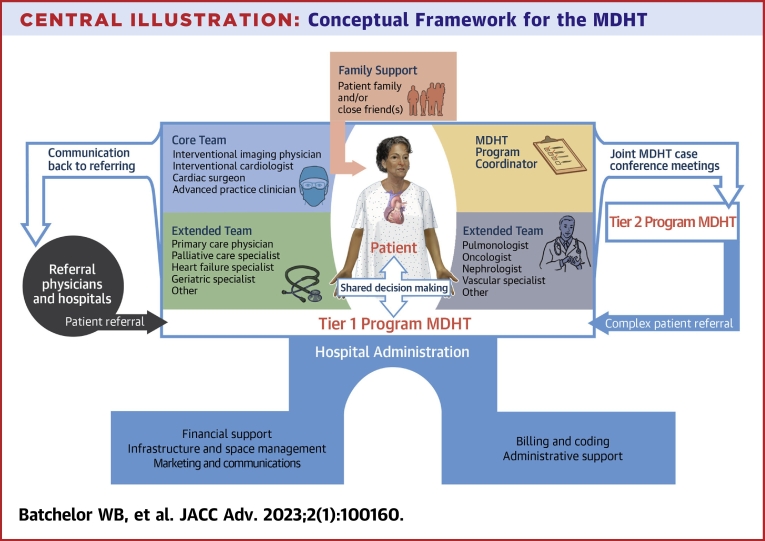
**Conceptual Framework for the MDHT** The figure depicts the relationships between the key entities involved in the multidisciplinary care of patients with complex cardiovascular disease. Typical members of the core and extended teams and responsibilities of hospital administration are shown. The **arrows** highlight the relationships and communication that exist between these entities. Tier 2 program multidisciplinary heart teams (MDHTs) may share the same internal components as a tier 1 program. The Central Illustration was completed through a collaboration of Devon Stuart (Devon Medical Art) and Mary Kate Wright (MK Illustrations).

## Contemporary role of the MDHT in clinical practice

Although best known for their role in VHD, cardiovascular MDHTs are routinely used in a wide range of clinical scenarios. To follow is a discussion of how MDHTs play an important role in: 1) VHD; 2) myocardial revascularization decisions (PCI vs CABG); 3) advanced HF and cardiac transplantation; 4) ACHD; 5) cardio-oncology; 6) cardio-obstetrics; and 7) geriatric cardiology. Clinical case examples that illustrate the role of the MDHT in these scenarios are presented in Table 1.

**Table 1:** Case Examples Depicting the Role of MDHT

Case Examples	Treatment Options	Recommended MDHT Members	Key MDHT Functions
A 78-y-old frail woman with type II DM who walks with a cane and presents with a non-STEMI, 3V CAD (Syntax score 32), acute on chronic stage III CKD, LVEF 40%, and “forgetfulness”	PCI vs CABG vs Med Rx	CC, IC, CTS, APC, GM, Neph	1. Assess medications, frailty, nutritional status, physical & cognitive functioning 2. Anatomic suitability for CABG vs PCI 3. SDM: CABG vs PCI vs Med Rx 4. Prevent/treat ARF before and after intervention
A 64-y-old man with symptomatic, severe Sievers I bicuspid AS, COPD, LVEF 45%, and an ascending thoracic aorta diameter of 4.4 cm who “prefers” TAVR.	TAVR vs SAVR	IC, CTS, ACI, APC, PMS	1. Assess COPD severity and optimize Rx 2. Assess bicuspid AV and raphe calcification, annulus size, and LVOT calcification 3. TAVR vs SAVR ± repair of TAA 4. TAVR: delineate long-term follow-up for TAA
A 52-y-old man with a history of remote mantle radiation for Hodgkin’s disease who presents with acute HF, LBBB, LVEF 30%, thrombocytopenia, and severe MR due to annular dilatation and mild P2 posterior leaflet mitral valve prolapse.	Mitral TEER vs Surgical MVr/MVR vs TMVR vs Med Rx	IC, EP, CTS, ACI, HFC, APC, Hem	1. Optimize GDMT and consider CRT-D 2. Determine MR etiology (primary vs secondary) and reassess severity after optimal GDMT and CRT-D 3. Assess suitability for TEER vs surgical MVr/MVR vs TMVR (within clinical study) 4. Determine cause of thrombocytopenia 5. SDM: decision on above MR therapies
An 81-y-old man with a history of treated multiple myeloma, primary (AL) cardiac amyloidosis, LVEF 30%, peripheral neuropathy, stage IV CKD and chronic NYHA III-IV HFrEF who presents with acute HF and LFLG gradient AS with AVA 1.0 cm^2^. CFS is 5.	TAVR vs Med Rx vs Hospice care	HFC, IC, ACI, CTS, GM, APC, Hem, PC, Neph	1. Assess medications, frailty, nutritional status, physical & cognitive functioning 2. Assess prognosis of MM/amyloidosis 3. Determine medical futility of TAVR (“Cohort C” patient?) 4. Determine severity of LFLG AS 5. SDM: Med Rx vs TAVR vs hospice care
A 63-y-old woman with a history of dilated CM (LVEDD 68 mm), CRT-D, severe secondary MR, and LVEF 25%, presenting with prolonged hypotension, ARF, recurrent hospitalization, jaundice, and elevated transaminases	Cardiac Tx vs TEER vs LVAD vs Med Rx	HFC, IC, CTS, ACI, APC, Neph, GI	1. Assess hemodynamics and need for inotropes, vasopressors, and/or MCS 2. Assess renal and hepatic function 3. Assess suitability for TEER 4. SDM: TEER vs LVAD vs cardiac Tx
A 31-y-old woman presenting G1P0 at 15 wks of gestation with severe rheumatic MS (MVA 1.0 cm^2^), moderate MR, PASP 65 mm Hg. NYHA II.	Pregnancy termination vs medical Rx vs BMV	IC, CTS, CC, ACI, card-obst, MFM	1. SDM: continuation of pregnancy vs termination 2. Determine anatomic suitability for BMV 3. Careful 3rd trimester and postpartum surveillance for HF
A 28-y-old male with hypoplastic left heart syndrome and 3 prior cardiac surgeries: Norwood, bi-directional Glenn and lateral tunnel Fontan. Presents with increasing fatigue, exertional dyspnea, and palpitations associated with worsening RV systolic function, moderately severe TR, and intermittent AF.	Fontan revision with Maze and valve repair vs cardiac Tx	ACHD team, HF, CTS, CA, SW, PC, psychologist	1. Assess RVEF with cardiac MRI 2. Assess liver function/cardiac cirrhosis 3. Assess renal function 4. Assess for protein losing enteropathy 5. Assess suitability for redo cardiac surgery 6. SDM: redo cardiac surgery vs Tx 7. Weigh short- and long-term outcomes
A 70-y-old woman with history of carcinoid syndrome presents with worsening fatigue, lower extremity swelling, abdominal bloating, stage III CKD, severe tricuspid regurgitation, moderate tricuspid stenosis and severe pulmonic regurgitation associated with right HF.	Surgical valve replacement (TV + PV) vs palliative/hospice care	Card-Onc, HFC, CTS, IC, CA, oncologist, ACI PC, Neph	1. Assess frailty, physical and cognitive functioning, and nutritional status 2. Determine extent of extracardiac carcinoid 3. Assess severity of right HF 4. SDM: surgical valve replacements vs Med Rx vs hospice care

ACHD = adult congenital heart disease; ACI = advanced cardiac interventional imaging physician; AF = atrial fibrillation; AL = amyloid light chain; APC = advanced practice clinician; ARF = acute renal failure; AS = aortic stenosis; AVA = aortic valve area; BMV = balloon mitral valvuloplasty; CA = cardiac anesthesiologist; CABG = coronary artery bypass graft; CAD = coronary artery disease; Card-Obst = cardio-obstetrics; Card-Onc = cardio-oncologist; CC = clinical cardiologist; CFS = clinical frailty scale; CKD = chronic kidney disease; CM = cardiomyopathy; COPD = chronic obstructive pulmonary disease; CRT-D = cardiac resynchronization therapy defibrillator; CTS = cardiothoracic surgery; DM = diabetes mellitus; Endo = endocrinologist; EP = cardiac electrophysiologist; GDMT = guideline directed medical therapy; GI = gastroenterologist; GM = geriatric medicine specialist; Hem = hematology; HFC = heart failure cardiologist; HFrEF = heart failure with reduced ejection fraction; IC = interventional cardiologist; LBBB = left bundle branch block; LFLG = low flow low gradient; LVAD = left ventricular assist device; LVEF = left ventricular ejection fraction; LVOT = left ventricular outflow tract; MCS = mechanical circulatory support; MDHT = multidisciplinary heart team; MFM = maternal fetal medicine specialist; MM = multiple myeloma; MR = mitral regurgitation; MRI = magnetic resonance imaging; MV = multivessel; MVR = mitral valve replacement; MVr = mitral valve repair; Neph = nephrologist; non-STEMI = non-ST-segment elevation myocardial infarction; NYHA = New York Heart Association; PASP = pulmonary arterial systolic pressure; PC = palliative care specialist; PCI = percutaneous coronary intervention; PMS = pulmonary medicine specialist; PV = pulmonic valve; RV = right ventricle; SAVR = surgical aortic valve replacement; SDM = shared decision-making; SW = social worker; TAA = thoracic aortic aneurysm; TAVR = transcatheter aortic valve replacement; TEER = transcatheter mitral valve edge-to-edge repair; TMVR = transcatheter mitral valve replacement; TR = tricuspid regurgitation; TV = tricuspid valve; Tx = transplant.

### Role of the MDHT in evaluating patients with VHD

As previously mentioned, the MDHT has become firmly embedded in the care of the VHD patient. The roles of the MDHT in this setting are to evaluate the patient’s severity of disease, determine which, if any, interventions are appropriate, and to discuss the risks, benefits, and alternatives of available treatment options with the patient.^11^^,^^16^ Thorough assessment of patients' symptoms and accurate interpretation of multimodality cardiac imaging and invasive hemodynamics are often necessary for the evaluation of patients with complex VHD. In this setting, optimal interpretation of cardiac imaging data often requires input from a structural heart interventional imaging physician. For patients in need of valve repair or replacement, the MDHT must formulate a recommendation for either transcatheter or surgical intervention. This process should take into account individual patient preferences and health values through the use of shared decision-making^17^ while satisfying the CMS criteria of being “reasonable and necessary”, which are required for payment.^11^^,^^16^

TAVR, the most frequently performed VHD procedure, is currently approved for low-, intermediate-, and high-risk patients with AS. Therefore, it is important for the MDHT to render treatment decisions across a broad spectrum of patient age, surgical risk, and anatomic scenarios (ie, patients with bicuspid disease, aortic root enlargement, and/or concomitant CAD). The MDHT must also identify older “Cohort C” TAVR candidates who may be too irreversibly compromised for AVR and better suited for hospice care.^18^ Consultative geriatric and/or palliative care specialists play an important role in this setting. The American College of Cardiology (ACC) has a useful decision aid that can help patients make informed decisions on surgical AVR vs TAVR. Similar concepts underpin MDHT decision-making in the care of patients with mitral, tricuspid, and pulmonic diseases.^11^ In the process of rendering treatment decisions, the lifelong management of the VHD patient must be carefully considered, including device durability and the feasibility and safety of future procedures.

Core team members of the MDHT for VHDs typically include interventional cardiologists, cardiac surgeons, structural heart interventional imaging physicians, and advanced practice clinicians (APCs).^8^^,^^11^ Although several other health care professionals may play key roles prior to, during, and/or after the hospital phase of care (ie, referring physicians, consulting cardiologists, cardiac anesthesiologists, medical and/or surgical subspecialists, nurses, cardiac catheterization laboratory and operating room staff, pharmacists, and clinical research coordinators), they are often not part of the core team.^11^ Specific case examples of how the MDHT plays a role in the care of the VHD patient are presented in Table 1.

### Role of the MDHT in myocardial revascularization decisions (PCI vs CABG)

With the prevalence of CAD increasing in the United States, cardiologists are seeing more patients with complex multivessel disease, including left main and/or triple vessel disease.^19^ Treatment decisions for these patients must consider a wide range of patient ages, comorbidities, procedural risks, and personal preferences. Technical advances and improved operator experience have made PCI a feasible option for more high-risk patients with multivessel and/or left main CAD, many of whom are at too high risk for CABG due to their advanced age or comorbidities. The 2021 ACC/American Heart Association/Society for Cardiovascular Angiography and Interventions coronary revascularization guidelines provide evidence-based treatment recommendations for patients with multivessel CAD and emphasize the importance of MDHT input.^20^ However, the evidence base underpinning these treatment recommendations stem from clinical trials comparing CABG to PCI that have generally excluded patients with severe comorbidities and marked frailty. It is in the space of uncertainty and clinical equipoise between PCI and CABG that the MDHT has its greatest value.^21^ When rendering revascularization recommendations for such patients, careful consideration of age, the technical barriers to CABG or PCI (vascular access/peripheral arterial disease, porcelain aorta, or hostile chest), and comorbidities (ie, severe chronic lung, kidney, or liver disease, active cancer, depressed left or right ventricular function, severe concomitant valvular disease, prior cardiac surgeries, cognitive impairment, and nutritional status) is paramount.^20^

For reviews of patients with high-risk complex CAD, core members of the MDHT typically include those directly involved in deciding on and performing coronary revascularization procedures (ie, interventional cardiologists, cardiac surgeons, cardiac anesthesiologists, and APCs). However, extended team members (ie, geriatric, palliative care, and/or HF specialists) may be necessary to provide additional input prior to or following revascularization. Institutions involved in the routine care of these complex patients may define specific operators (interventional cardiologists and cardiac surgeons) who are proficient in performing technically challenging, high-risk coronary revascularization procedures. A fully operational complex CAD MDHT should provide formal review of cases by a team that at least includes both interventional cardiologists and cardiac surgeons.^22^ Pertinent clinical information, including symptoms, medical/surgical history, laboratory findings, noninvasive testing (transthoracic echocardiography, stress test), and invasive data (right and left heart catheterization, coronary angiogram), is best captured on a case report form that may be used during MDHT meetings to communicate pertinent information and facilitate final treatment recommendations.^22^ Thus far, the widespread use of the MDHT in this setting has not been achieved, with a recent report from a large academic medical center showing that only 3% of potential patients were referred for MDHT review.^22^ Specific case examples for which the MDHT may help determine the optimal management of high-risk patients with complex CAD are shown in Table 1.

### Role of the MDHT in advanced HF and cardiac transplantation

Despite the advances in pharmacological and device-based therapies, the outcomes of patients with advanced HF remain poor.^23^ With treatment options becoming increasingly complex, multidisciplinary team-based care is necessary to deliver effective therapies across the spectrum of HF patients. Multidisciplinary HF care has been shown to improve the overall quality of care, patient engagement, and medication safety, while reducing the duration and frequency of recurrent HF hospitalizations.^24^, ^25^, ^26^, ^27^ The recently published ACC/American Heart Association/Heart Failure Society of America HF guidelines endorse multidisciplinary care to assist in the transition from the inpatient to outpatient setting and to reduce the risk of rehospitalization; recommendations that also apply to patients receiving heart transplantation and left ventricular assist devices.^28^ Regulatory agencies and payers, including the Organ Procurement and Transplantation Network, The Joint Commission, and CMS, have mandated a multidisciplinary team approach for the evaluation and care of advanced HF patients undergoing left ventricular assist device placement or heart transplant surgery.^29^

In the past decade, there have been significant advances in pharmacologic, transcatheter, and surgical therapies for HF. Although several new therapies have become commercially available, not all have been included in the most recent treatment guidelines.^28^ Therefore, MDHT input is often necessary to provide insight into when and how to deploy the most up-to-date and effective advanced therapies, while recognizing medical futility. The core members of the MDHT for advanced HF patients typically include advanced HF cardiologists, APCs, pharmacists, nurses, and HF program coordinators. Extended MDHT members may include critical care physicians, cardiac surgeons, physical therapists, palliative care specialists, nephrologists, endocrinologists, pulmonologists, social workers, dieticians, transplant and/or left ventricular assist device nurses, infectious disease specialists, and psychologists. Team member input varies depending on the clinical setting (ambulatory vs hospital), specific needs and social circumstances of the patient, and local expertise. The recent emergence of effective treatments for infiltrative cardiomyopathies, such as cardiac amyloid and sarcoid, has placed further emphasis on the MDHT as treatment often requires input from a variety of health care providers (ie, cardiologists, advanced cardiac imaging physicians, pharmacists, geneticists, pulmonologists, and endocrinologists). The rapidly expanding fields of structural and device-based HF therapies also require effective care coordination between HF specialists, interventional cardiologists, cardiac surgeons, interventional imaging physicians, and intensivists. When rendering treatment plans, the multidisciplinary HF team must incorporate patient preferences and health values while striving to reduce health care disparities.^30^ Specific case examples illustrating the role of the MDHT in treating patients with advanced HF are shown in Table 1.

### Role of the MDHT in ACHD

Patients with major CHD defects often require specialized health care for the entirety of their lives. Historically, care for these patients has tended to be disjointed, typified by multiple individual providers delivering care from within silos, resulting in fractured care. In 2017, the Adult Congenital Heart Association launched an accreditation system that defined the resources, staff, and processes necessary for the optimal care, clinical outcomes, and safety of ACHD patients. Two levels of programs were identified: care centers and comprehensive care centers. The former work with the latter more advanced centers as satellite programs. Currently, there are 43 accredited CHD programs in the United States; the MDHT is a critical component in each of these programs. Core members of the ACHD MDHT often include ACHD medical directors, cardiac surgeons (with specialized training in CHD), interventional cardiologists, electrophysiologists, HF specialists, cardiac anesthesiologists, APCs, ACHD nurses, and social workers. To adequately address the complex needs of ACHD patients, the MDHT should also have access to reproductive medicine services, advanced imaging, and treatment for pulmonary arterial hypertension, thereby drawing pulmonary medicine, obstetrics and gynecology, and imaging specialists into the extended team. In putting forth the final treatment recommendations, it is important for the MDHT to consider the lifelong treatment plan for ACHD patients and follow their long-term outcomes. Specific examples in which the MDHT plays a role in the care of patients with ACHD are presented in Table 1.

### Role of the MDHT in cardio-oncology

Cardiovascular disease and cancer share common risk factors, including older age, health behaviors, and comorbidities, which help explain their frequent coexistence.^31^ Certain cancer therapies and malignancies, themselves, play an etiologic role in the development and progression of cardiovascular diseases. This has led to the need for cardiovascular professionals specifically trained to manage patients with active cancer and cancer survivors with existing cardiovascular disease, under the umbrella of cardio-oncology. Cardio-oncologists have expertise in providing comprehensive care to patients with co-existing heart disease and cancer and in preventing and treating the cardiovascular complications of cancer and cancer therapies, with an aim to improve patient prognosis and limit interruptions in cancer therapy.

Cardio-oncology requires multidisciplinary cooperation among cardiology, hematology-oncology teams, and a variety of other disciplines. Cardiologists, oncologists, and APCs form the primary core members of cardio-oncology MDHTs, and pharmacists play a key role in helping form cardiac and oncologic treatment plans with the aim of minimizing the risk of drug-drug interactions, QTc prolongation, bleeding, and thromboembolism. However, the composition of the MDHT and timing of cardiac procedures may vary according to the stage of cancer, timing and nature of cancer therapy, and the urgency for, and need of, cardiac intervention. For example, patients with carcinoid VHD typically require a MDHT comprised of cardiologists, cardiothoracic surgeons, cardiac anesthesiologists, and medical oncologists to evaluate the patient's cardiovascular and functional status, extent of extracardiac carcinoid disease, and to guide the use of somatostatin analogues before, during, and after surgery to prevent perioperative carcinoid crisis.^32^^,^^33^

**Figure 1:**
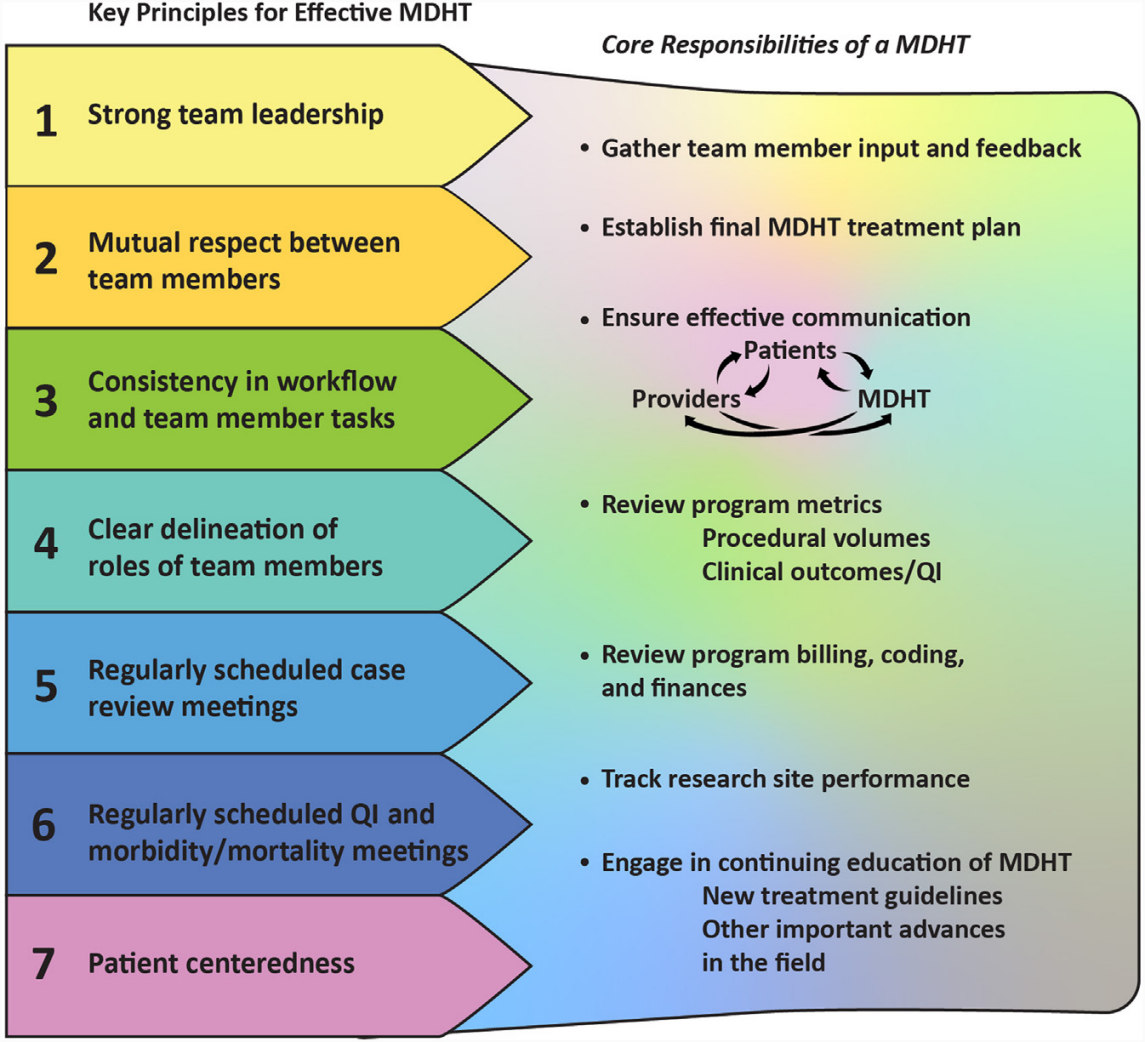
**Key Principles for the Effective Operation of the MDHT and its Core Responsibilities** MDHT = multidisciplinary heart team; QI = quality improvement.

The International Cardiac Tumor Board serves as an example of an effective multidisciplinary working model designed to serve an uncommon group of cardiovascular malignancies.^34^ This tumor board has shared leadership and collaboration from cardiac surgery, medical oncology, cardiology, radiation oncology, imaging, and pathology across several institutions in the United States, Canada, and Europe. Monthly hybrid virtual and onsite tumor board meetings are coordinated by APCs, and the board is set up for case presentation, discussion, and referral to tertiary oncologic and surgical institutions. This quaternary care model serves multiple institutions across several countries and fits into the conceptual framework of the MDHT illustrated in Figure 1. A case example of how the MDHT plays a role in the care of cardio-oncology patients is presented in Table 1.

### Role of the MDHT in cardio-obstetrics

The United States is the only industrialized nation facing increasing maternal mortality, for which cardiovascular disease is the leading cause, and Black and Hispanic mothers are affected disproportionately.^35^^,^^36^ The hemodynamic changes during pregnancy, including increases in heart rate and cardiac output, may exacerbate preexisting CVD conditions.^37^ Cardiovascular diseases that occur preconception, during pregnancy, or in the peripartum phase may require the expertise of an MDHT. Examples of chronic cardiovascular diseases that significantly impact pregnancy include stable CAD, VHD (ie, mitral and AS in particular), and cardiomyopathies (ie, peripartum cardiomyopathy). Several urgent cardiovascular scenarios, including acute myocardial infarction resulting from spontaneous coronary artery dissection, hemodynamic compromise from VHD in the third trimester and postpartum phases, and cardiogenic shock secondary to peripartum or other cardiomyopathies, may present abruptly and require emergent care. Patients with aortopathies, including those associated with a bicuspid aortic valve disease, also require close monitoring and MDHT care during the stages of pregnancy.

The prenatal cardio-obstetric evaluation typically includes a thorough review of the patient’s past medical history, prior cardiovascular procedures, medication history and need for adjustment during pregnancy, and exercise stress testing in patients with asymptomatic VHD.^38^ Genetic counseling may be indicated in patients with heritable conditions such as aortopathies. Evaluation from a cardiac surgeon and/or an interventional cardiologist on the MDHT is warranted in patients with severe VHD to determine which, if any, intervention is indicated preemptively to avoid decompensation during pregnancy. Multidisciplinary care may also be warranted prior to conception for risk assessment and consideration of preconception interventions.

The goal of the cardio-obstetrics MDHT is to maximize both maternal and fetal health. Therefore, a unique challenge facing the MDHT is to formulate recommendations that are not only patient-centered but also consider fetal/neonatal health. Expansion of the MDHT to include specialists in maternal fetal medicine and neonatology is often required to assess fetal risk relative to gestational age. Because cardiac surgery during pregnancy is associated with an increased risk of fetal loss, percutaneous transcatheter therapies are often preferred when feasible and effective.^39^ However, the effects of ionizing radiation on the unborn fetus need to be taken into consideration, and efforts should be made to minimize radiation exposure to both mother and fetus during these procedures. Case examples illustrating the role of the MDHT in cardio-obstetrics are presented in Table 1.

### Role of the MDHT in geriatric cardiology

The U.S. population is rapidly aging. In the year 2034, older adults (age 65+ years) are projected to outnumber children (<18 years old).^40^ Aging is often accompanied by changes in physical and cognitive function, frailty, sarcopenia, multimorbidity, increased risk of falls, disturbances in urinary function, and reduced life expectancy.^41^^,^^42^ Because the majority of older TAVR patients have sarcopenia^43^ and more than a quarter continue to have functionally limiting symptoms after valve replacement, geriatric consultation may be useful in selected patients, either preceding or following TAVR. Treatment decisions in older frail adults require that health care professionals, patients, families, and caregivers come to a clear understanding of a patient’s baseline status, procedural risks vs benefits, personal preferences, health values, and prognosis, both with and without intervention.^11^^,^^16^^,^^44^ The 2020 ACC Expert Consensus Decision Pathway for TAVR calls for the routine pre-TAVR assessment of frailty, disability, physical and cognitive function, and procedural futility.^11^ Patients with baseline major impairment within these domains should be referred for comprehensive geriatric evaluation.^45^ A detailed geriatric evaluation helps the MDHT understand the degree to which an older frail patient may achieve enough improvement in symptoms, functional status, quality of life, and/or survival to justify procedural risks. In older adult patients with HF, interdisciplinary geriatric care has been associated with improved quality of life and spiritual well-being, and reduced anxiety and depression.^46^ During the COVID-19 pandemic, the expansion of digital health (telehealth, telemedicine, mobile health, and remote patient monitoring), internet access, and cellular technologies have provided further opportunity to enhance care and improve health outcomes for older adults.^47^ Case examples of how the MDHT plays a role in the management of the older adult with cardiovascular disease are presented in Table 1.

## What is the evidence in support of the MDHT?

Despite the routine use of the MDHT in clinical practice, its effectiveness in improving patient outcomes has been poorly studied. The evidence underpinning its Class I recommendation has been based solely on nonrandomized, observational studies and consensus opinion (Level of Evidence: C).^11^^,^^48^ The use of a multidisciplinary team has been associated with improved survival in patients with invasive breast cancer.^49^ Reductions in variability of care, adherence to standardized protocols, and multidisciplinary expertise are posited reasons for benefit in that setting. A systematic overview of 7 randomized controlled trials reporting the impact of acute care multidisciplinary team intervention on hospitalized older patients showed reductions in emergency department readmission rates, mortality, and functional decline.^50^ In the realm of cardiovascular disease, the registry of Emory Angioplasty vs Surgery Trial demonstrated that the final coronary revascularization strategy agreed upon by the MDHT produced better survival than that noted in the randomized trial cohort.^51^ A recent retrospective analysis of 3,399 patients undergoing TAVR in a South Australian tertiary care center reported a 20% reduction in 5-year risk-adjusted mortality following the implementation of an MDHT.^52^ In the SYNTAX III REVOLUTION Trial, the MDHT was firmly rooted into the clinical trial design itself, playing a central role in evaluating the value of coronary computed tomography angiography compared with conventional angiography in the management of multivessel CAD.^53^ These findings, from predominantly observational studies, reveal an association between MDHT care and improved patient outcomes but fall short of proving causality.

It should also be recognized that MDHT evaluation itself may create challenges, including the potential for discordant recommendations.^54^ When the care of 237 patients with multivessel CAD was independently reviewed by 8 different blinded heart teams, discordant treatment recommendations were observed in nearly a third.^54^ Greater discordance was noted for patients treated with PCI and medical management than for those treated with CABG. This is important because discordant recommendations may cause confusion in patients and/or erode trust. In summary, although the MDHT has become firmly embedded in comprehensive heart programs across the United States, the evidence supporting its use is based on observational studies. Therefore, future investigation is needed to elucidate what, if any, benefit is conferred by MDHT care on patients (ie, clinical outcomes, quality of life, patient satisfaction) and the heart team itself (ie, team member engagement and satisfaction, team efficiency, agreement/discordance, and productivity).

## Operationalizing the MDHT

The core responsibilities of the MDHT are to: 1) gather team member input and feedback; 2) establish the final MDHT treatment plan; 3) ensure effective communication between MDHT, patients, and providers; 4) review program metrics (procedural volumes and clinical outcomes/quality improvement processes); 5) review program billing, coding, and finances; 6) track research site performance; and 7) ensure that team members are kept up to date with treatment guidelines and/or other relevant developments in the field (Figure 1). We believe there are 7 key principles for the effective operation of the cardiovascular MDHT (Figure 1), several of which have been promulgated by the National Academy of Sciences.^55^ Defining the independent roles and duties of each team member is critical, and there should be clarity around who is responsible for presenting cases during MDHT meetings. We recommend that the meeting agenda and all pertinent patient clinical data be circulated to MDHT members prior to the meeting and available throughout the meeting. Routine use of a standardized case report to collect key data elements may be useful. The hospital administration should help support the MDHT by managing infrastructure, staffing, coding/billing, resource allocation, and marketing/advertising of the program (Central Illustration).^11^ Regularly scheduled MDHT meetings are necessary to gather team members, review clinical data, reach consensus, and finalize treatment recommendations. Weekly meetings are generally sufficient for this purpose, although meeting frequency will vary depending on clinical case load and member availability. Meetings, either in-person and/or virtual (ie, video conferencing), may also serve to review program metrics, procedural outcomes, accuracy of documentation, coding and billing, clinical research protocols, and referral networks. We also recommend that MDHTs engage in regularly scheduled morbidity and mortality conferences, from which quality-improvement initiatives may be recognized and launched, and patient care processes and safety reviewed.^56^ Although there is no widely accepted standard on how MDHTs render decisions, consensus opinion is often used. We recommend that each MDHT establish a process for making decisions, dealing with disagreement, and communicating with patients and referring providers.

## Challenges facing the MDHT

There are several challenges with incorporating and maintaining a MDHT. First, the need for patients to be seen by several specialists and undergo multiple diagnostic tests places significant physical, psychological, and financial burdens on patients and caregivers. The scheduling challenges associated with bringing together various team members on a regular basis represent another limitation. Many MDHTs convene weekly clinics, where patients with specific cardiovascular conditions are seen by all relevant subspecialists during the same visit. The benefits of this paradigm include patient convenience and the potential for more rapid decision-making. However, logistics may be problematic, and efficiency is not optimal. Premeeting discussions among team members can help address specific clinical questions prior to the official meeting, thereby improving meeting flow and efficiency. A team member (or members), often the program coordinator, should be tasked with setting the meeting agenda and collecting, organizing, and presenting relevant clinical information. Adequate hospital resources and meeting space are needed; however, the resources available in a large academic institution may not be present in smaller, low-volume programs. Still, the key principles and goals of MDHT meetings (Figure 1) should be adhered to, regardless of clinical setting.

Many programs may not have the collective experience and medical subspecialization to address the broad array of complex clinical scenarios. In this setting, there may be value in forming a partnership with a larger more experienced high-volume tier 1 program (Central Illustration). This may be accommodated through videoconferencing for case presentations and electronic sharing of imaging data. Such a relationship may help triage complex patients to the more advanced tier 1 program, while building experience and expertise in the tier 2 center. Such networks of care may also afford patients access to more advanced treatment options than otherwise available in their local community. In this setting, effective communication, both among clinicians and between clinicians and patients, is paramount. Keeping the patient fully informed and central to the process helps ensure shared decision-making.^16^^,^^57^ Another pervasive challenge for MDHTs is to reduce health care disparities related to race, gender, ethnicity, age, rurality, and social determinants of health such that they do not prevent patients who are in need of care from accessing and receiving it.^58^ An understanding of the demographics of the patients cared for within the MDHT relative to the surrounding community may provide insights into these disparities, allowing for interventions to minimize treatment gaps.^36^^,^^58^ Currently, substantial variability exists in the composition and operation of MDHTs across the United States and other countries. Similar to the multidisciplinary organ transplantation and cancer care models, the MDHT paradigm could benefit from greater standardization of staffing models, implementation guidelines, training opportunities, and measurement of outcomes. Finally, there exists no reimbursement structure from payors that assigns monetary value to the time providers spend engaged in MDHT meetings and other patient care-related activities.

## Study limitations

The ideas and recommendations put forth in this perspective are from the views and opinions of the authors. Our goal is to put forth a conceptual framework for the MDHT and its contemporary role in treating patients across a broad range of cardiovascular disciplines and clinical scenarios. This review does not describe the various instruments that may be used to measure successful functioning of MDHTs. For this, the reader should refer to literature from multidisciplinary cancer teams.^59^ Given the lack of experimental evidence underpinning our claims on the optimal composition, structure, and functioning of cardiovascular MDHTs, the recommendations and opinions put forth are based on expert opinion and a limited number of observational studies.

## Conclusions

The MDHT plays a central role in contemporary care models for a wide range of cardiovascular diseases. The value of the MDHT lies in its ability to transcend the limitations of established care guidelines by providing the most up-to-date and informed treatment recommendations for complex individual patients through the consolidation of multiple team member inputs. The major challenges facing the MDHT include the increasing number of clinical scenarios that require its input, a lack of widespread subspecialty expertise, time constraints of team members, and a lack of reimbursement structure that assigns value to provider time engaged in MDHT activities. Although MDHT care has become routine in the treatment of patients with complex CAD, VHD, and ACHD and in the fields of cardio-obstetrics, cardio-oncology, and geriatric cardiology, there remains a lack of experimental data confirming improved patient outcomes. Therefore, future investigations are needed to define: 1) best practices for MDHTs; 2) which disease scenarios benefit most from their input; and 3) to what extent MDHT care improves the effectiveness of treatment decision-making, team member efficiency/satisfaction, and ultimately patient outcomes. Notwithstanding these limitations, the MDHT will remain a critical component for the successful delivery of cardiovascular care well into the foreseeable future.

## Funding support and author disclosures

Dr Batchelor has received institutional research grant support from Abbott and Boston Scientific; and is a consultant for Abbott, Medtronic, Edwards, V-Wave Medical, and Boston Scientific. Dr Anwaruddin is a consultant/proctor/advisory board member at Medtronic; proctor at Edwards; on the steering committee of Boston Scientific; on the advisory board of OpSens; and has equity in East End Medical. Dr Wang is a consultant at Edwards Lifesciences, Boston Scientific, Abbott, and Neochord; and has received institutional research grant support from Boston scientific. Dr Velugapudi is on the Speakers Bureau of Abiomed and Opsens; and is on the advisory board of Women’s Health Initiative. Ms Wyman is a consultant at Edwards Lifesciences and Boston Scientific. Ms Perpetua is a consultant at Abbott and Edwards; and is on the advisory board of Abbott. Dr Maini has equity in East End Medical; is on the advisory board of Boston Scientific, Abbott, and Medtronic; is on the Speakers Bureau of Boston Scientific, Abbott, and Medtronic; and has received proctorship honoraria from Boston Scientific, Abbott, and Medtronic. Dr Mack is a co-principal investigator/study chair (no renumeration) for Abbott, Edwards Lifesciences, and Medtronic. Dr Leon is on the advisory board (no renumeration) of Abbott, BSC, MDT, Edwards, Gore, and Venus Medtech. All other authors have reported that they have no relationships relevant to the contents of this paper to disclose.
